# 36-Month Evaluation of a Weight Management Programme in Chinese Overweight and Obese Adults

**DOI:** 10.3389/fpubh.2021.749302

**Published:** 2021-10-21

**Authors:** Xi Yang, Kaushik Chattopadhyay, Richard Hubbard, Jia-Lin Li, Li Li, Yi Lin

**Affiliations:** ^1^Department of Nutrition, Ningbo First Hospital, Ningbo, China; ^2^Division of Epidemiology and Public Health, School of Medicine, University of Nottingham, Nottingham, United Kingdom; ^3^Department of Endocrinology and Metabolism, Ningbo First Hospital, Ningbo, China; ^4^Center for Health Economics, Faculty of Humanities and Social Sciences, University of Nottingham, Ningbo, China

**Keywords:** weight management, weight loss, weight maintenance, overweight, obesity, China

## Abstract

**Background:** Few comprehensive lifestyle intervention programmes have been investigated on overweight and obese adults in China. This study was to evaluate the effect of a 36-month weight management programme on weight loss and its maintenance among overweight and obese patients in Ningbo, China.

**Methods:** Adults with BMI ≥24*kg*/*m*^2^ enrolled in this programme, including nutritional, physical activity, psychological and endocrinological counselling sessions, from July 2015 to January 2020. Adults participated in face-to-face counselling sessions and group-based education. Then, participants joined 21-day intensive programme using Bohe health APP and WeChat group to get personal advice of nutrition and lifestyle. In the end, participants were requested to join 33-month follow-ups including face-to-face counselling and personal advice on WeChat group. The main outcome was to evaluate the changes in weight at each followup from baseline weight.

**Results:** In total, 692 adults participated in this entire weight management programme. During follow-ups, 579, 475, 299, 219, and 135 adults participated at 3, 6, 12, 24, and 36 months, respectively. All participants had a significant initial weight loss at 3 months, then maintained the weight loss during 33-month follow-ups. At 36 months, 11.0%, 6.4%, and 3.5% of all participants achieved 5%, 10%, and 15% weight loss from the baseline weight, respectively. Adjusted weight at 36 months was significantly reduced from the baseline weight in both sex (−7.2 kg).

**Conclusion:** This weight management programme is suggested to benefit to reduce initial body weight and maintain long-term weight loss among overweight and obese adults.

## Introduction

Obesity is a worldwide epidemic (1). Obesity is defined as an excessive accumulation of body fat which causes significant comorbidities, including cardiovascular disease (CVD), type 2 diabetes (T2D), hypertension, dyslipidemia, certain types of cancer, and mortality (2–4). With rapid economic development, industrialisation and urbanisation for the past four decades, the determinants of overweight and obesity in China are the changes of dietary pattern and lifestyle. The prevalence of overweight and obesity in Chinese adults dramatically increased from 9.1% (5) and 2.0% (6) in 1989 to 41.3% and 15.7% in 2015 (7), respectively, thus, overweight and obesity have become one of important Chinese public health concerns.

Weight loss is an important way to decrease the prevalence of obesity and the risks of obesity-related chronic diseases. Evidence showed that short-term weight-loss intervention programme focusing on promotion of lifestyle involving nutrition and physical activity can result in clinically significant health benefits (8, 9). However, weight maintenance is a major challenge for weight loss in overweight and obese individuals. Recently, lifestyle intervention through strategies was suggested that higher autonomous motivation, self-efficacy, and self-regulation skills emerged might contribute to long-term weight control and weight loss maintenance (10).

Ningbo is one of the most economically developed cities in Zhejiang Province, which is located in the richest region of Yangtze River Delta, China. Recent research studies proved that the high prevalence of overweight and obesity in Ningbo (11, 12) was close to the national prevalence (7, 13), and reached similar prevalence of 20 European countries (14) and global prevalence (1).

To date, few comprehensive lifestyle intervention programmes have been investigated in overweight and obese adults in China. This weight management intervention programme was developed based on autonomous motivation and self-regulation focusing on long-term weight loss and weight control. Our previous 6-month feasibility study suggested that the lifestyle intervention strategy could benefit to weight treatment in overweight and obese patients (15). The objective of the present study was to evaluate the effectiveness of the 36-month comprehensive lifestyle intervention programme on weight loss and maintenance of weight loss in overweight and obese adults in Ningbo.

## Methods

### Study Design and Setting

This study is a pragmatic evaluation of an ongoing weight management programme delivered in real life supported by Ningbo government. In total, 977 patients from July 2015 to January 2020, who were invited and willing to participate in this 36-month programme during outpatient counselling visits at Department of the Endocrinology of Ningbo First Hospital, Zhejiang Province, China. All adults aged 18–75 years with body mass index (BMI) ≥24 kg/m^2^ were included in this programme (16). The exclusion criteria of this programme included: (1) age <18 years at baseline; (2) secondary obesity caused by other medical conditions or diseases; (3) diagnosis of any types of cancer or severe coronary heart disease; (4) receiving any related non-pharmaceutical/pharmaceutical intervention; (5) pregnancy or lactation; (6) mental illness; (7) cognitive impairment. The target goal of this programme is to achieve 5–15% weight loss in the overweight and obese patients.

The ongoing research programme is approved by the Ethics Committee of Ningbo First Hospital, China (No. 2019-R049). Written informed consents were obtained from all the participants.

### Measurement of Baseline

All the eligible participants were asked to complete dietary behaviour and lifestyle questionnaires including socio-demography, dietary intakes for the past week, physical activity for the past week, medication record and medical history. Besides that, participants were asked to fill out questionnaire of Yale Food Addiction Scale (YFAS) to identify the addiction of their usual dietary intakes (17).

Health status including anthropometry, blood pressure and biomarkers was examined by experienced nurses. Anthropometry, including height, weight, waist circumference, hip circumference, basal metabolic rate, body fat percentage and muscle mass percentage, was examined by body composition detector (GAIA KIKA, Korea) (18). Blood pressure was measured using an electronic sphygmomanometer on the right or left arm after a 5-min rest. Biomarkers containing blood lipids (total cholesterol, triglyceride, low-density lipoprotein cholesterol, high-density lipoprotein cholesterol), fasting plasma glucose, fasting plasma insulin, fasting c-peptide, glycated haemoglobin A1c, uric acid, alanine aminotransferase, aspartate aminotransferase, and free fatty acids were examined by biochemical automatic analyzer.

### Measurement of Follow-Ups

The body weight and height were measured at 3, 6, 12, 24, and 36-month follow-ups, after completing the same questionnaires in baseline.

### Intervention

The weight management programme including nutrition, physical activity, psychology and endocrinology delivered by a multidisciplinary team consisting of three clinicians, two dieticians, two exercise specialists, and one psychologist (Table 1). All the participants were divided into 10-person groups and assigned to the intervention programme on group basis. One group leader was selected by the group members, who was a key factor to support our multidisciplinary team. The function of the group leaders was to motivate his/her members participating the rest of follow-ups and reminded them to record their daily dietary intake, physical activities and changes in their lifestyle. At the baseline visit, each individual participated in face-to-face counselling sessions for personal guidance according to the medical reports (Table 1). The purpose of nutritional advice was to reduce energy-dense foods by controlling diet portion size and regulating dietary intakes rationally. During the nutritional counselling session, each participant was advised to adjust their dietary patterns based on the distribution of daily energy intake from macronutrients and change dietary patterns appropriately (19). During the counselling session of physical activity, participants were motivated to join outdoor and indoor physical activity at conveniences, guided the appropriate types of physical activity and encouraged to exercise at least 150 min per week. The advice on physical activity followed the American College of Sports Medicine (ACSM)'s guideline for exercise testing and prescription tenth edition for overweight and obese adults (20). Participants at psychological counselling session were asked to report their recent mood status (happiness, satisfaction, self-esteem, depression stress, anxiety, and sleep quality) and their behaviour changes in their recent daily life were discussed with clinical psychologist. Participants were suggested to record their behaviour changes and mood status, and document the progress. During the endocrinology session, advice on medication and was delivered based on their baseline health examination. After face-to-face counselling sessions, all the participants as group unit gathered together for education programmes delivered by one general physican.

**Table 1:** Multidisciplinary weight management programme.

**Phase**	**Time**	**Programme**	**Individual/group**	**Duration**
1	1st month	Face-to-face counselling	Individual	1 h
1	1st month	Face-to-face education	Group	2 h
2	21-day/1st month	Intensive programme Synchronous online (Bohe health APP on mobile)/WeChat	Individual	10 min
3	3–36 month	Face-to-face counselling	Individual	2 h
3	3–36 month	Synchronous online (Bohe health APP on mobile)/WeChat	Individual	10 min

All the participants recorded their daily dietary intakes and physical activity via Bohe health APP on mobile. Bohe health APP was created for personal management to evaluate dietary patterns, nutrition status and anthropometric status (e.g., body weight) (21). Besides APP online programme, participant group basis joined WeChat group (22), organised by the multidisciplinary team. All adults participated in a 21-day intensive intervention programme (phase 2). During phase 2, participants were compulsory to report their anthropometry (e.g., body weight), daily dietary intakes and physical activity to the multidisciplinary team through WeChat. Self-measured body weight was suggested to participants reporting on WeChat online programme in order to motivate participants to self-regulate and self-control their body weight. At the WeChat online counselling session, participants got their personal feedbacks on personal advice, their doubts of weight management, performance, and progress.

During the follow-ups, advice on nutrition and physical activity was delivered face to face to provide their personal solution (phase 3). At the psychological counselling session, participants gained professional advice if they had questions and desired to have a chat with clinical psychologists. Moreover, participants on group basis followed synchronous online (Bohe health APP on mobile) and WeChat for further advice as well when they had doubts and questions. Each patient got personal feedback from our professional multidisciplinary team.

### Parameters and Outcomes

#### Obesity Definition

Measured body weight was collected at Ningbo First Hospital to evaluate their weight loss during the follow-ups. BMI, used as a measure of obesity, was calculated as weight (kg)/height (m^2^). Participants were classified into four BMI categories according to China Obesity Task Force (COTF) as follows: underweight (<18.5 kg/m^2^), normal weight (18.5–23.9 kg/m^2^), overweight (24.0–27.9 kg/m^2^), and obesity (≥ 28.0 kg/m^2^) (16). Central obesity was defined according to WC values: WC >90 cm in men or >85 cm in women (16).

#### Socio-Demographic Status

Participants were asked to fill out a standard questionnaire about their socio-demographic status, designed and validated by the Ningbo First Hospital. Education were categorised into three levels including lower secondary education; vocational, technical or high school; higher education (bachelor, master, or above). Participants provided their marital status (Single/divorce/widowed/separated, married, and unknown status).

### Statistical Analysis

Descriptive analysis was presented as number and percentage for category variables and mean and standard deviation (SD) or median for continuous variables. Statistical differences in age and biomarker parameters were compared between men and women by Student's *t*-test and Mann-Whitney *U* test. A sensitivity analysis was undertaken for comparison between participants' baseline weight and baseline weight among those dropout participants at 36 months aiming to detect whether missing values of dropouts affect the outcomes. Mean changes in weight across 36-month study period controlling for age, education levels and marriage status were examined with Bonferroni correction by repeated measures analysis of covariance. Mean weight loss at 36 month between men and women was tested by non-parametric test.

Results were considered statistically significant at a two-tailed level of 0.05. Statistical analysis were conducted using the STATA statistical software package version 15 (2017).

## Results

### Attendance of Intervention and Follow-Ups

In total, 692 out of 977 adults (40.8% men) fulfilling all inclusion criteria and willing to participate in this 36-month programme were included in the baseline (Table 2). Among all the enrolled patients, 579, 465, 290, 204, and 122 patients participated in the follow-ups at 3, 6, 12, 24, and 36 months, respectively.

**Table 2:** Recruitment and follow-up of participants.

	**Total (*n*)**	**Male (%)**
Recruited participants	977	40.9
Baseline	692	40.8
3 month	579	39.9
6 month	475	37.5
1 year	299	33.8
2 year	219	33.3
3 year	135	30.4

### Baseline Characteristics of Participants

The baseline characteristics of participants was described in Table 3. The mean age of all the participants was 30.9 years (men: 29.4 years, women: 31.9 years). Mean weight and BMI were significant higher in men than women. Around 87.6% and 95.7% of participants were defined to have general obesity and abdominal obesity, respectively. The result of sensitivity analysis showed that no significant difference of baseline weight between all participants and dropout participants at 36 months (*P* = 0.678).

**Table 3:** Baseline characteristics of participants (2015–2020).

	**Total** **(*n* = 692)**	**Men** **(*n* = 282)**	**Women** **(*n* = 410)**
Age (year)	30.0	30.0	28.4^*^
Weight (kg)	93.8 (19.2)	106.5 (18.3)	85.0 (14.3)^*^
BMI (kg/m^2^)	33.5 (5.1)	34.7 (5.0)	32.6 (5.0)^*^
	*n* (%)		
Education			
No education or lower secondary	53 (7.7)	11 (3.9)	42 (10.2)
Vocational, technical, or upper secondary school	162 (23.4)	72 (25.5)	90 (22.0)
Higher education	477 (68.9)	199 (70.6)	278 (67.8)
Marital status			
Single/divorce/widowed/separated	321 (46.4)	157 (55.7)	164 (40.0)
Married	363 (52.5)	121 (42.9)	242 (59.0)
Unknown	8 (1.2)	4 (1.4)	4 (0.98)
Overweight	86 (12.4)	14 (16.2)	72 (86.0)
Obesity	606 (87.6)	268 (44.2)	338 (55.8)
Abdominal obesity^a^	651 (95.7)	272 (41.8)	379 (58.2)

*Data represent mean with standard deviation or median*.

a *The number of patients was 680*.

* *Mean differences between men and women (P <0.001)*.

### Weight Loss

Weight loss was evaluated across the entire 36-month programme (Table 4). Weight significantly reduced after 36 months in both sex with and without Bonferroni correction (adjusted difference, male: −7.0, *P* < 0.001; female: −7.4, *P* < 0.001). Participants had a significant initial weight loss of adjusted weight at 3-month follow-up in both sex. Mean weight at each follow-up was significant from mean baseline weight. However, adjusted weights were slightly regained in both sex (adjusted weight: men, −7.4 kg at 24 months and −7.0 kg at 36 months; women, −7.4 kg at 36 months).

**Table 4:** Adjusted weight change (kg) from baseline.

**Gender**	**3 month**	**6 month**	**12 month**	**24 month**	**36 month**
Total	−5.2 (0.360)^a^	−6.9 (0.530)^a^, ^b^	−7.6 (0.705)^a^, ^b^	−7.5 (0.800)^a^, ^c^	−7.2 (0.881)^a^
Men	−5.9 (0.679)^a^	−7.0 (1.1)^a^	−7.8 (1.2)^a^	−7.4 (1.1)^a^	−7.0 (1.3)^a^
Women	−4.9 (0.408)^a^	−6.8 (0.621)^a^, ^b^	−7.5 (0.893)^a^, ^c^	−7.6 (1.1)^a^	−7.4 (1.1)^a^

*Values of weight changes are mean with standard error*.

^*^Difference in weight changes across follow-up periods was examined with Bonferroni correction for multiple comparisons by repeated measures analysis of covariance.

a *Mean weight difference from baseline weight (P < 0.001), with Bonferroni correction for multiple comparisons*.

b *Mean weight difference from 3-month weight (P < 0.001), with Bonferroni correction for multiple comparisons*.

c *Mean difference from 3-month weight (P < 0.05), with Bonferroni correction for multiple comparisons*.

**Figure 1:**
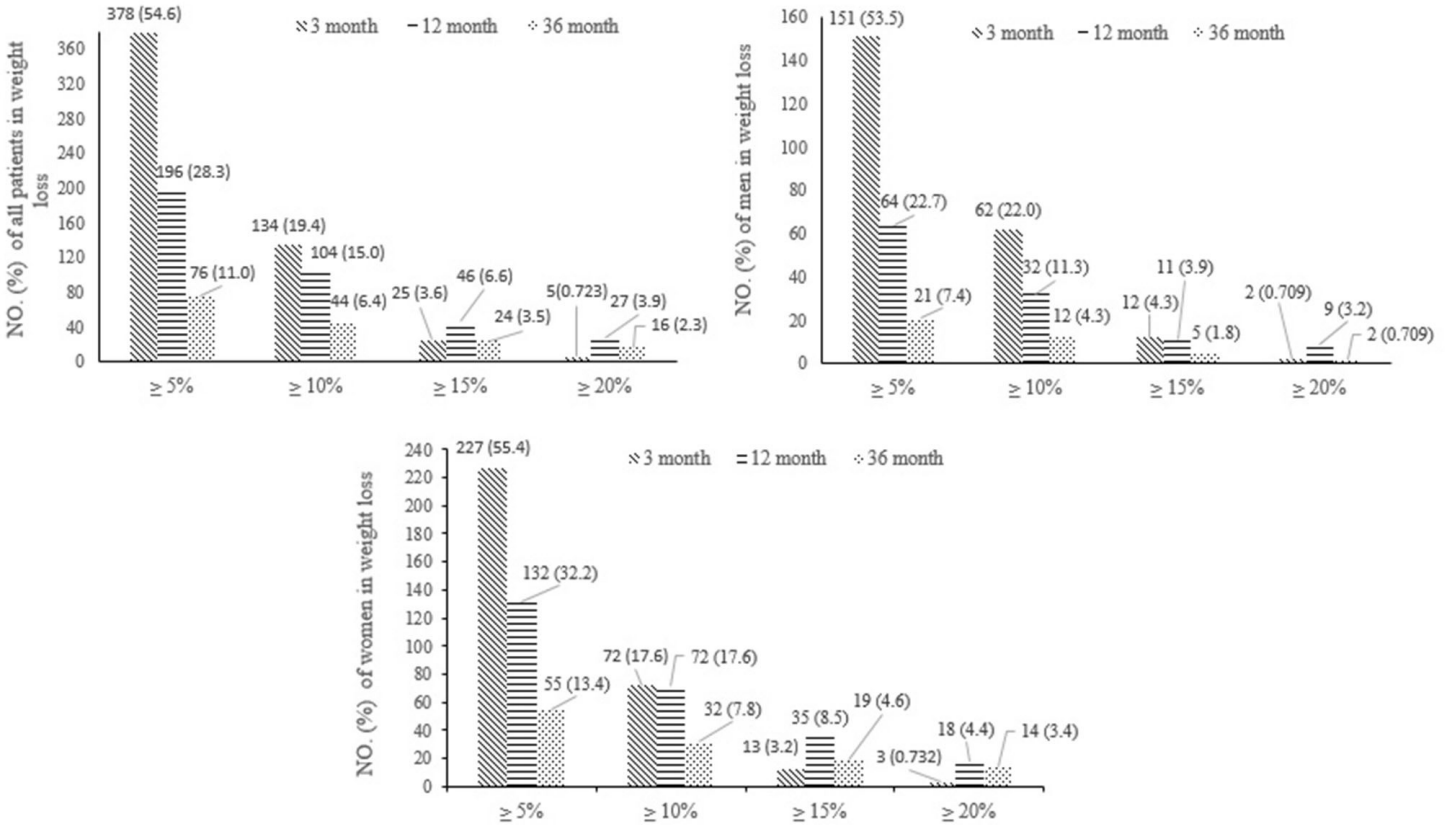
Effects of interventions on weight loss in total 692 participants from the baseline weight at 3, 12, and 36-month follow-ups.

A sizable proportion of participants sustained clinically significant weight loss at 3, 12, and 36-month follow-ups (Figure 1). Overall, at 36-month follow-up, 8.7% of all baseline participants (6.7% of men and 10.0% of women) reached the target goal of weight loss 5–15%. Moreover, 2.3% of all baseline participants (2 men and 14 women) reached ≥20% weight loss at 36 months, exceeding the originally target goal.

## Discussion

To all authors' knowledge, the present study was the first long-term comprehensive lifestyle intervention to evaluate the effect of weight management programme on weight loss and its maintenance in overweight and obese outpatients in Ningbo. Our weight managment programme, focusing on regulating dietary intakes and lifestyle, and promoting positive life attitude and behaviour, was to address reducing body weight and maintaining weight loss. The results after the 36-month programme demonstrated the significant benefits to weight loss in both sex.

In the present study, a mean adjusted weight of 7.0 kg and 7.4 kg was reduced in both men and women, respectively, after 36 months. In our previous feasibility study, BMI was observed to reduce by 2.4 kg/m^2^ after 6 months (15). Lifestyle intervention including nutritional counselling and physical activity was evidenced to be more effective on weight loss in overweight and obese people (23–25). However, a big challenge in weight management of obesity is maintaining weight loss. Evidence stated that long-term success rates of lifestyle weight loss treatments are so low and may be fruitless (25, 26). In the present study, mean adjusted weight at 36 months was much lower than baseline weight. At the end of our programme, 6.7% of men and 10.0% of all participanting women maintained weight loss and reached the target goal of weight loss 5–15%. Even, 2 male and 14 female patients got weight loss ≥20%. Therefore, the weight management programme can be effective and clinical benefits to obese patients.

Most lifestyle intervention trials on overweight and obese adults failed due to rapid weight regain after an initial weight loss intervention period (25, 27). A 12-week weight reduction programme including dietary advice and physical activity regimes indicated a regain of 1.39 kg/m^2^ BMI after initial 12-week weight loss (25). Weight regain was observed in our study as well. Nevertheless, an important difference is that, unlike other lifestyle intervention, weight regain was almost in the end of our study and weight regain was minor. This might be due to intensive intervention programme (phase 2), keeping counselling sessions and frequent feedbacks via WeChat (phase 3). Personal solution and advice with psychological and social supports to our patients might be critical to contribute to maintenance of change in eating behaviour and positive lifestyle during 36-month period (28). Our previous feasibility study in line with our findings indicated to keep reducing BMI at 3 and 6-month follow-ups, respectively (15). While, a previous lifestyle intervention reported no significant difference between intensive counselling and short-term counselling interventions (28).

An important key to progress in weight loss is the effective method. In this regard, an individual and group strategy including face-to-face counselling and interactive technology-based intervention (e.g., WeChat group) was used in our intervention and 33-month follow-ups. Thus, this weight management strategy can enhance adherence in weight management programme through efficient and frequent communication, which can increase the success rate of this entire study. A recent systematic review and meta-analysis, supporting our weight management strategy, suggested an extended care is essential and efficacious to address long-term maintenance of lost weight (−3.2 kg) (29). Therefore, our intensive intervention can prevent weight regain after initial weight loss and our phase 3 can maintain long-term weight loss.

Comparing to one previous Trials of Hypertension Prevention Phase II (TOHP-II), our study and TOHP-II both used similar weight management strategy including individual counselling session and group meetings focusing on dietary intakes, physical activity, and social support (30). An additionally similar strategy for both studies is an intensive lifestyle intervention followed by less intensive intervention programme. The result of in TOHP-II showed that mean weight change from baseline was −0.2 kg at 36 months, which is far less than weight loss in our study. The significant difference is that our strategy included psychological counselling session to provide emotion, psychological and mental support. A meta-analysis including 36 randomised controlled trials indicated that psychological interventions that combined with dietary and physical activity strategies was more useful to enhance weight reduction (−4.9 kg) (31).

Women had a slight more weight loss at 36 months than men, although no significant difference was found. In addition, more almost 10% female patients reached our original weight loss target (5–15%) and 12 more women exceeded our target than men. Most weight management intervention studies were conducted to compare weight loss between intervention group and control group. The rate of weight loss success can be attribute to the different characteristics between men and women in our study. Physiological mechanisms including higher plasma leptin concentrations in women (32) and more percentage of muscle mass in men (33) may result in the different level of weight loss between men and women. Attendance in this study indicated that women were more eager to lose body weight for keeping healthy and desired body shape (34).

### Strengths and Limitations

Our study is the first comprehensive lifestyle counselling intervention programme to evaluate the effect of our strategies on weight loss and maintenance of weight loss. Our progrmme strategies included individual counselling and group education, provided personal feedback through WeChat APP during the entire programme. However, this programme still has some limitations. First, the complicated intervention design with long-term follow-up resulted in high dropouts at 36 months indicating participants lost interests in this long-term programme. Relocation of our participants and switching hospitals caused high dropouts as well. Second, self-reported body weight used for their personal feedbacks via WeChat online sessions might not reflect the real body weight of overweight and obese patients due to psychological factor. The under-reported body weight cannot affect the accurate weight and patients could not gain appropriate feedbacks and advice resulting in the influence in weight control and weight loss maintenance. Third, due to the structure of our questionnaires, some basic information was not well-collected, thus, baseline study could not reflect more details on daily energy intake and physical activity level. The last but not least is that no control group can be compared to the intervention group because all the outpatients followed the same standard intervention programme in this ongoing real life study. In the future study, energy intake and physical activity should be well-collected to estimate the levels for total energy intake and physical activity, which may affect weight loss.

## Conclusion

The results indicated that overweight and obese participants got significant initital weight loss after 3 months and maintained wight loss during 33-month follow-ups. Therefore, this comprehensive lifestyle intervention programme is suggested to benefit to long-term weight management in overweight and obese people. In the future, the effectiveness of individual interventions on overweight and obese people needs to be investigated to compare with control group in a randomised control trial.

## Data Availability Statement

The raw data supporting the conclusions of this article will be made available by the authors, without undue reservation.

## Ethics Statement

The studies involving human participants were reviewed and approved by the Research Ethics Committee, Ningbo First Hospital. The patients/participants provided their written informed consent to participate in this study.

## Author Contributions

YL performed and interpreted statistical analysis and drafted manuscript writing. XY was responsible for data collection, quality control, and manuscript preparation. KC, RH, and J-LL supported manuscript writing. LL and YL contributed to the study design for the whole research. All authors read and approved the final manuscript.

## Funding

This study was supported by the Natural Science Foundation of Ningbo (Grant no. 2016A610169) and Ningbo Technology Innovation 2025 Major Special Project, Study on the global intelligent management of diabetes mellitus and the cellular immune mechanism of vascular diseases (Grant no. 2019C50094).

## Conflict of Interest

The authors declare that the research was conducted in the absence of any commercial or financial relationships that could be construed as a potential conflict of interest.

## Publisher's Note

All claims expressed in this article are solely those of the authors and do not necessarily represent those of their affiliated organizations, or those of the publisher, the editors and the reviewers. Any product that may be evaluated in this article, or claim that may be made by its manufacturer, is not guaranteed or endorsed by the publisher.
